# Optimal and scalable entanglement distribution over crossbar quantum networks

**DOI:** 10.1038/s41598-024-62274-x

**Published:** 2024-05-22

**Authors:** Bogdan-Călin Ciobanu, Luca Perju Verzotti, Pantelimon George Popescu

**Affiliations:** Computer Science and Engineering Department, University POLITEHNICA of Bucharest, 60042 Bucharest, Romania

**Keywords:** Electrical and electronic engineering, Quantum information

## Abstract

Crossbar networks are a cornerstone of network architectures, capable of operating both as standalone interconnections or as integral switching components in complex, multi-stage systems. The main advantages of crossbar networks are their non-blocking operation and unparalleled minimal latency. With the advent of large scale quantum networks, crossbars might be an important asset towards the Quantum Internet. This study proposes a solution for the problem of distributing entanglement within crossbar quantum networks. Entangled particles are a consumable resource in quantum networks, and are being used by most quantum protocols. By ensuring that nodes within quantum networks are being supplied with entanglement, the reliability and efficiency of the network is maintained. By providing an efficient, scalable framework that can be used to achieve optimal entanglement distribution within crossbar quantum networks, this study offers a theoretical achievement which can be also used for enhancing quantum network performance. An algorithm for selecting an optimal entanglement distribution configuration is proposed and fully tested on realistic possible configurations.

## Introduction

Entanglement^1–3^ represents a uniquely quantum phenomena exhibited by systems of particles, and is an essential ingredient for quantum communications^4^. Entangled pairs (e-pairs) enable efficient distributed quantum computing^5–8^, and secure communication via quantum key distribution^9,10^ and quantum teleportation^11–14^. The preservation of entanglement fidelity^15^ is paramount in quantum communications; it hinges on the system’s ability to remain isolated from any environmental interaction. This necessity poses a significant challenge: while e-pairs must be shielded from external influences to maintain high fidelity for transmissions over long distances, they also require precise manipulation and measurement.

The subject of this delicate balancing act are usually photons with polarization-encoded quantum information^16–19^. For quantum networks, these represent essential, yet exhaustible resources which are consumed during fundamental operations such as quantum teleportation and other protocols. As such, if communicating parties require reliable, sustained transmissions, it is crucial to establish an entanglement resupply system across the network^20–22^. The most straightforward method would be to physically transport a particle of an e-pair to each of the two nodes in need. This technique significantly reduces entanglement fidelity, as the entangled particles, traveling at best half the distance between nodes, given that the e-pair was generated at an intermediary node midway between the two parties, are susceptible to loss and environmental interference in the lossy transmission mediums^23–25^. This approach, however, may not be compatible with the extensive network sizes anticipated for the Quantum Internet^26–29^. The Quantum Internet is envisioned as a global network that interconnects heterogeneous quantum networks, demanding a more scalable solution.

Interconnection networks^30,31^ have a pivotal role in the realm of classical computing and communications, acting as the backbone for data exchange and system integration. In the context of quantum technologies, the importance of interconnection networks is bound to be at least as significant. The interconnections of quantum systems^32^ are tasked with not just the transfer of quantum information, but also with the preservation and manipulation of quantum states. Their development and optimization thus stand as a cornerstone in realizing the full potential of quantum communications and computing. Crossbar interconnection networks in particular hold significant potential due to their unique properties, like their ability to create direct and dynamic connections between inputs and outputs, which can greatly enhance the efficiency of information transmission. This is crucial in quantum computing environments where the rapid and reliable exchange of quantum states is essential.

The objective of this work is to propose and analyze the optimal strategy for distributing entanglement over crossbar quantum networks, as multiple concurrent entanglement requests would have to share the nodes and links of the interconnection network. The paper is structured as follows. In Section “Classical interconnection networks” an overview of classical interconnection networks is presented. Through Section “Challenges of concurrent entanglement distribution” entanglement distribution schemes are explored, and solutions for a basic entanglement distribution setup are provided. Section “Optimal entanglement distribution in crossbar quantum networks” describes the optimal method for choosing the entanglement distribution configuration over a crossbar quantum network. In Section “Simulation results and scalability analysis”, an explicit algorithm, simulation results and interpretations are presented. The paper ends with conclusions regarding the optimality and scalability of the presented entanglement distribution algorithm.

## Classical interconnection networks

In the context of communication networks, a cornerstone of performance is the seamless and efficient transfer of data between communicating parties. The concept of interconnection networks represents the backbone of data transfer. Interconnection networks (interconnects) are complex topological structures that facilitate data transmission between multiple components. More precisely, an interconnection network is a structured arrangement of communication links and switches, designed to ensure efficient data exchange, minimize latency, or maximize throughput, depending on the chosen interconnection architecture.

Historically speaking, interconnection networks were used in telecommunication networks, namely telephone, to connect individual lines without the need of manual patching. Since then, the principles of interconnection have been adopted into broader applications, such as parallel and grid computing^33^, optical networks^34^, and network switching fabrics of the Internet.

The defining characteristic of an interconnection network is its topology. Each topology is designed to suit specific needs and scenarios, and comes with its own advantages and drawbacks^31^. Such topologies include Linear Arrays and Rings, which are among the simplest, with network components connected in a straight line or loop, respectively. These can offer a simple way to route data, but limited scalability. Meshes and Toruses expand upon these topologies, branching out in two or three dimensions, creating grid-like structures, hence improving flexibility. Hypercube topologies are a binary n-dimensional construct, which has a logarithmic diameter, with respect to its number of nodes. Crossbar networks offer direct connections between every input and output, and stand out for their non-blocking nature, although, in practical implementation, this advantage comes with the cost of being resource-intensive in terms of implementation.

In this work, we will focus on the crossbar topology, as it offers unparalleled non-blocking communication, due to its direct connectivity between components. Furthermore, it offers deterministic low latency communication, which is uniform and minimal across all communication scenarios. As this work is purely theoretical, we will not consider the material costs of implementing crossbar networks, although we acknowledge that it is possible they can be high, compared to alternative topologies. However, in Section “Simulation results and scalability analysis”, we analyze how our proposed solution might scale by limiting the number of resources needed to interconnect vast amounts of nodes, through the well-known Benes^35^ network topology.

As envisioned use cases of crossbar quantum networks for entanglement distribution, such networks may be deployed in order to facilitate quantum communication between arrays of quantum computers or quantum memories. Alternatively, these networks could be used to create a distributed quantum sensing infrastructure, where entanglement would be used in communication between nodes. One other use case for such a crossbar quantum network could be in the backbone of the Quantum Internet. Functions of the Quantum Internet could include applications which have no classical counterpart such as unconditionally secure communications^36^, or quantum teleportation networks.

### Crossbar networks

**Figure 1:**
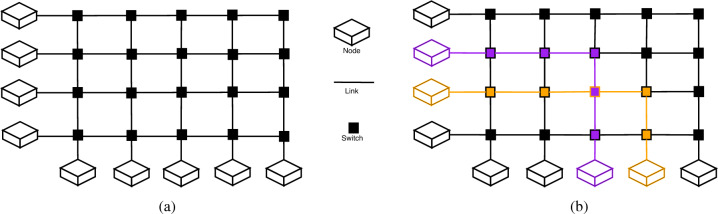
(**a**) represents a crossbar network topology, and its constituent elements. By convention, on the left side we have the inputs, and at the bottom the outputs. In (**b**) an example of how routing is performed between inputs and outputs within a crossbar. It should be noted that the number of inputs does not necessarily need to match the number of outputs.

A crossbar can be defined as a network with N inputs and M outputs, which can serve at most $min\{N, M\}$ connections concurrently, without having dedicated channels between each input and output. The crossbar consists of shared switching elements that forward the traffic through the network, connected by shared links, as can be seen in Fig. 1a. However, as we have alluded to, this kind of topology is faced with a scalability problem. For an $N \times M$ crossbar, a total of $N \times M$ switching elements need to be deployed along $N + M$ links.

Traffic routing essentially determines how network resources (links and switches) are assigned for message transmissions. Traffic routing through a crossbar network is fairly straightforward. As we can see in Fig. 1b, to send a message from an input, the traffic needs to flow along the row of the input until the row intersects with the column of the output. There, the corresponding switch will route the traffic along the column, until it reaches the output node. This scheme ensures non-blocking communication, regardless of the chosen permutation of inputs and outputs.

Besides scalability in terms of implementation cost, large-scale systems which want to leverage crossbar switches face issues regarding signal integrity due to longer links, and multiple switches placed on the same link. These factors can also create crosstalk or longer propagation delays, potentially affecting the performance and reliability of the interconnection network. A way to mitigate these problems is to use smaller crossbar networks as building blocks for larger, multistage interconnection networks. In multistage interconnection networks traffic needs to pass through multiple crossbar switches before reaching the destination node. Usually, all the crossbar switches used have the same size, and the size of the crossbars alongside the number of stages of the entire interconnection determine different properties of the network. Some examples of such multistage interconnection networks are the previously mentioned, Benes network topologies^35^, Clos networks^37^, and modern topologies such as the Scalable Crossbar Network^38^.

### Components of crossbar quantum networks

The realm of quantum computing introduces many complex and intriguing concepts. Envisioning a quantum analog of classical crossbar interconnection networks, we can reach a grid-like architecture of intermediary nodes, connected through links. As this is a quantum network, the aim of such an interconnection network is not to transfer classical information through packets, but rather to transfer quantum information through physical particles.

Quantum information is encoded in a Quantum Bit (qubit). Information that is meant to be transferred along long distances is typically encoded in photons, by means of their polarization, due to several reasons, first of which is the robustness against decoherence that photons exhibit. Inherently, the quantum states are very fragile, and are susceptible to decoherence due to environmental factors. Photons are less sensitive to these interactions compared to other carriers such as more massive particles. This means that they can be used for long distance communication, not only because they travel at the speed of light, but also because they do so with minimal loss of coherence while traveling through the medium. Modern telecommunication networks rely on fiber-optic networks, and polarization-encoded quantum states can be transmitted through these existing links, provided a dark fiber channel is allocated^39^. Lastly, polarization states of photons can be easily prepared, manipulated and measured using commercially available optical elements. This makes it relatively straightforward to perform quantum operations on polarization-encoded qubits.

The links, as stated before, can be optical fiber links, or they can be Free-Space Optical (FSO) - transmission of photons through open air or vacuum. It should be noted that each type of link comes with its own advantages and disadvantages. It should be noted that, regardless of the medium chosen, signal amplification is not possible due to the no-cloning theorem^40^. As such, we need to envision systems where the same photon which left one of the communicating parties is the same photon received by the other party.

Typically, optical fiber links exhibit lower decoherence for the same distance as an FSO link. Longest fiber link channel through which quantum information was transmitted has 830km between nodes^41^, while the record for a terrestrial FSO link currently is of up to tens of kilometers^25^. This is due to the fact that the atmosphere exhibits fluctuations of its refractive index, which leads to light scattering, diffraction or beam wandering. These effects disturb the quantum state, leading to decoherence. Furthermore, FSO communication is affected by background light (sunlight or artificial sources), as these introduce noise in the quantum state. While error correction procedures have been developed for quantum communications, typically these require a minimal state of coherence to work. While optical fiber exhibits effects such as birefringeance or non-linear effects, these are generally more predictable and have a lesser effect compared to the challenges of FSO transmissions.

An advantage of FSO is that transmissions can be made between a stationary and a moving target, for example, a satellite. Satellite-based quantum communication has achieved significant milestones, with qubits being transmitted between Beijing and Vienna. This is possible do to the fact that the drawbacks associated to terrestrial FSO transmissions - scattering, diffraction or beam wandering, are mostly negated in transmissions above the atmosphere, as these were caused by interactions with air molecules. There has been a significant scientific focus in the area of satellite-based quantum networks as a result of this fact.

The intermediary nodes, should act as the switches of the classical crossbar network. These nodes should be able to perform basic qubit manipulation, as well as being able to generate or measure qubits. Advanced methods such as entanglement purification^42,43^ may be employed within the nodes in order to maximize the fidelity of the entangled pair shared by the two communicating parties at the end. It is of note that entanglement purification typically requires a minimal fidelity in order to function, and works best with e-pairs that already have a high fidelity.

The functional capabilities of the switches (the intermediary nodes) align with the theoretical framework of quantum repeaters^44–48^, particularly in their ability to execute Bell State Measurements. This foundational operation is critical for quantum entanglement distribution networks, and is the minimum requirement of functionality of the intermediary nodes in the hereby presented scheme. Incorporating fully-fledged quantum repeaters with entanglement purification capabilities into the system architecture would theoretically enhance the entanglement fidelity across all stages of quantum state distribution, therefore increasing the terminal entanglement fidelity between network nodes.

However, if we are to consider quantum repeaters which can somehow boost the fidelity of entangled photons passing through them (either through entanglement purification or other techniques), we need to consider two possibilities. In case all the links of the crossbar quantum network have an equal number of quantum repeaters along them, such a setup would be perfectly compatible with the proposed scheme. In case there is an unequal number of repeaters within the network, we need to take into account that the repeaters introduce a delay in the transmission. Therefore, a mechanism of storing entangled particles received early until the counterpart from the other link is received needs to be available. This could be achieved by using quantum memories^49,50^. We will discuss the use of quantum memories in more detail in Section “Challenges of concurrent entanglement distribution”.

## Challenges of concurrent entanglement distribution

The possible functions of the Quantum Internet, as diverse as they may be, all have in common the need for entangled particles. These entangled particles are essentials to protocols such as quantum teleportation and quantum key distribution, as they effectively act as a medium of information transmission that is secured by physical laws.

**Figure 2:**
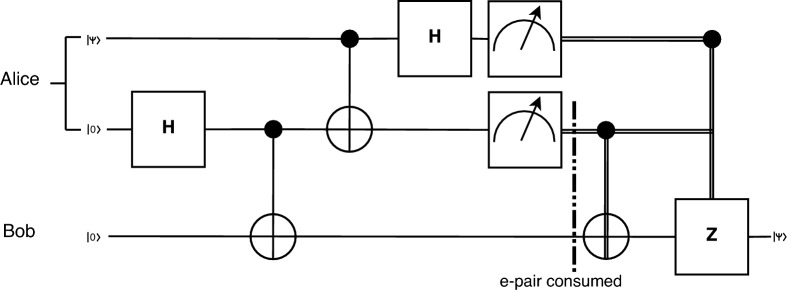
Quantum teleportation circuit. Alice begins with a qubit in the $|\psi>$ state. After acquiring an entangled pair, shared between Alice and Bob, and Alice performing a partial measurement, using 2 classical bits of information, Bob can retrieve the exact state that Alice began with, $|\psi>$.

The quantum teleportation protocol enables pairs of communicating parties to exchange quantum information without actually physically moving any quantum states during the course of the communication from one party to the other. This is done by leveraging the peculiar properties of entangled particles, particularly the fact that modifying the state of one of the particles in the entangled pair affects the other particle in a predictable manner, regardless of the space separation between the two particles. This is, however, not a superluminal transfer of information, as 2 bits of classical information need to be transmitted to the receiver, in order to retrieve the correct quantum state that the sender encoded, as it can be seen in the quantum circuit for teleportation, in Fig. 2. During the teleportation procedure, the entangled pair is consumed, (i.e. it is measured), and cannot be reused to perform another information transfer. This means that, for sustained quantum communication between parties by means of quantum teleportation, a way to reliably distribute entangled pairs needs to be in place.

### Existing entanglement distribution protocols

The entanglement distribution protocol detailed in the recent study by Yin et al.^51^ represents a significant leap in the pursuit of global quantum communication networks. The protocol proposed harnesses the advantages of satellite platforms to mitigate the signal loss inherent in terrestrial fiber networks, which has been a major bottleneck for long-distance quantum entanglement. By generating entangled photons on a satellite and distributing them to ground stations, the researchers have circumvented the limitations posed by Earth’s atmosphere and curvature. The high-fidelity entanglement achieved across distances of up to 2400 km is particularly notable, demonstrating that quantum correlations can be maintained over scales far exceeding traditional fiber-based systems. The approach benefited from cutting-edge technologies, including narrow-beam divergence to reduce photon dispersion and dynamic polarization compensation to counteract the alterations caused by the satellite’s motion and atmospheric interference.

In a recent analysis by Viscardi et al. ^52^ a sophisticated framework for optimizing the distribution of quantum entanglement, a foundational process in the emerging Quantum Internet, is presented. The entanglement distribution challenge is approached by formulating it as a Markov Decision Process, which enables the modeling of decision-making in quantum network nodes, for effective entanglement distribution. The model distinguishes between fixed parameters that depend on the underlying quantum network technology, such as the time horizon for distribution and the probability of successful entangled pair transmission, and design parameters that can be manipulated to achieve desired outcomes, primarily through the reward function. This reward function is pivotal in guiding the decision-making process, ensuring larger distributed cluster sizes and shorter distribution times.

The Markov Decision Process-based model is concluded to be a valuable tool for quantum network designers, providing a method to adjust and control the entanglement distribution process to meet specific performance benchmarks. This work not only aids in the practical engineering of quantum networks but also contributes to the foundational infrastructure necessary for realizing the Quantum Internet, with implications for secure communications and distributed quantum computing.

Another recent work focused on optimal entanglement distribution over star-shaped topologies^53^. In this work, two primary distribution methodologies have been introduced: symmetrical and asymmetrical approaches. These relate to the way intermediary nodes are allocated for intersecting routes. In order to perform the entanglement distribution within a single transfer cycle, each route can either do it’s measurements on odd-numbered nodes, and entangled pair generation on even-numbered, or vice versa. This choice influences the success probability of establishing entanglement between the two terminal nodes.

The findings of this study suggest that the choice of entanglement distribution strategy should be adaptive, depending upon the network’s transmission success probabilities, and the complexity of its topology. For example, networks with less reliable transmissions should use the symmetrical approach, where intersecting routes contend over the same intersecting node, is preferable. Alternatively, networks with highly reliable quantum operations should use an asymmetrical approach, which provides simultaneous resolution to multiple entanglement requests, but execute more measurements.

### Simple entanglement distribution between two nodes

In order to describe how we can distribute entanglement through a crossbar quantum network, we first need to define the purpose of the inner nodes of the crossbar. As stated before, these should be able to perform basic qubit manipulation. Namely, they should be able to perform Bell State Measurements (BSM), and be able to generate new entangled pairs.

The purpose of being able to perform Bell State Measurements is to be able to execute the entanglement swapping procedure^54,55^. Through entanglement swapping, we can effectively make two separate parties share an entangled pair without actually having to physically transport an entangled particle all the way from one party to the other. In the following, we shall consider a chain topology, with each party at the ends of the chain. An ideal application of entanglement swapping would be if two parties supply an entangled particle to a node that is situated at half the distance between the two of them. The node would then perform a BSM on the two particles thus shifting the entanglement to the two particles with whom they were each previously entangled to.

The advantage of such a distribution scheme relies in preserving entanglement fidelity. As the quantum state coherence is affected by environmental factors, so too is the entanglement fidelity of entangled particle pairs. Therefore, by exposing the state a shorter time to external factors, we preserve more of the initial entanglement fidelity. In the example application for entanglement swapping, we are effectively halving the distance the particles need to travel, and fidelity loss scales exponentially with the distance traveled. Thus, we not only end up with a higher fidelity entangled pair in the end, but we are doing so two times faster than by transporting the particle between the two parties (for which we will use a common naming convention - Alice and Bob).

An alternative solution to efficiently transmit an entangled pair to Alice and Bob would be to have a node midway between them. This midway node would generate an entangled pair, and transmit one particle to Alice, and one to Bob. Since the act of measurement itself is imperfect, and can fail, it is natural that we want to minimize such operations in our process to distribute entanglement. Compared to the previous distribution scheme, this one does not involve any measurements, only the entangled pair generation and two transmissions, each with half the distance between Alice and Bob. Therefore, this distribution scheme where we are not performing an entanglement swap is preferable.

It is noteworthy that in these solutions, and in the algorithm proposed in the following sections, we do not consider the usage of a quantum memory. The hereby proposed scheme is a solution where entanglement is generated and transmitted in the least amount of time possible, such that quantum state coherency is exposed to as few environmental factors as possible. Typically, classical crossbar networks are employed in small scales, where the transmission time between nodes is negligible. If, however, we wish to employ a crossbar quantum network over large distances, we would need to account for this transmission time, as well as clock drifts between nodes, and therefore use quantum memories to store entangled particles for the Bell State Measurements.

Regarding the capabilities of the quantum memories used, the coherence time of these quantum memories should be expected to be minimally equivalent to the duration needed for a photon to traverse the network’s longest link. If we were to consider longer coherence times for the quantum memories, such that entanglement could be preserved between the operation cycles of the crossbar, there may be more optimal solutions for the distribution of the entanglement. Furthermore, if the capacity of the quantum memories allows for more than one qubit to be stored, this may allow more sophisticated distribution schemes. We consider these scenarios to be open problems in the realm of entanglement distribution. Regarding the fidelity of the quantum memories, ideally these should be as high as possible. However, we acknowledge that quantum state coherence degradation might occur over time in quantum memories, and a trade-off might occur when choosing to serve a stored entangled qubit instead of generating a new one. This aspect should be taken into account in any further scenario that leverages quantum memories.

## Optimal entanglement distribution in crossbar quantum networks

The previously mentioned scenarios only involve three nodes: Alice, Bob, and a midway node which either performs a BSM, or generates entanglement. Transmissions of entanglement are limited by the distance that the photon can travel, as we have stated before. Thus, if Alice and Bob are at a long distance, multiple nodes might need to be placed between them in order to facilitate transmission of quantum information. In this situation, we need to adapt the aforementioned methods to the situation where there are multiple intermediary nodes between Alice and Bob.

### Efficient entanglement distribution along a chain topology

**Figure 3:**
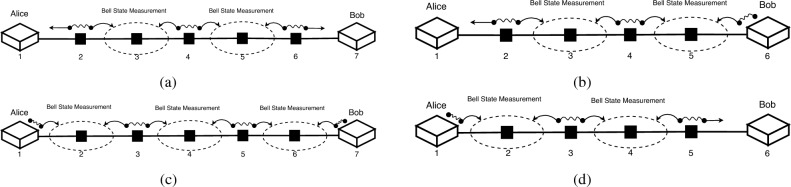
Four efficient entanglement distribution possibilities along a chain topology. In (**a**), there is an odd number of nodes, and we use an odd distribution scheme: nodes 3 and 5 perform the BSMs. Alternatively, in (**b**), the process of odd distribution is depicted along an even number of nodes, with nodes 3 and 5 performing the BSMs as well. In (**c**), the less efficient even distribution is used along an odd number of nodes, where nodes 2, 4, and 6 perform the BSMs, and in (**d**), the even distribution is used along an even number of nodes, with only nodes 2 and 4 performing the BSMs.

For both distribution schemes, the simplest way to adapt to multiple intermediary nodes would be to just use the additional nodes as proxies, and still use the midway node as a BSM machine, or as the generator of entanglement. However, these approaches don’t solve the problem of distance, as at least one particle would still have to travel more than half the distance between Alice and Bob. A solution would be to alternate between the two aforementioned distribution methods in such a way that no photon would need to pass through more than one link. In Fig. 3, we can observe the ways we can alternate these schemes to form what we will refer to even and odd distribution schemes. An even distribution scheme is one where even-numbered nodes (counting from Alice) generate entanglement, which they transmit bidirectionally towards their adjacent nodes (which are odd-numbered). The odd-numbered nodes then perform Bell State Measurements of these entangled particles. In Fig. 3c and d we can observe such distributions of entanglement where nodes numbered 3 and 5 perform the BSMs. Alternatively, in the odd distribution scheme, as in Fig. 3a and b, odd-numbered nodes generate entanglement, and even-numbered nodes perform the entanglement swapping procedure (nodes 2, 4 and 6 in Fig. 3a, and nodes 2 and 4 in Fig. 3b).

The benefits of such an alternating distribution scheme are manifold. For once, the particles have to travel at most the distance of the longest link in the chain. This would lead to a lower fidelity loss of the entanglement. Another result of this fact is that the entire operation would take as much time as it takes for a photon to travel the distance of the longest link, plus the time it takes to perform the BSM. This would also positively affect the final fidelity of the entangled pair shared by Alice and Bob. Furthermore, we are fully utilizing the qubit-manipulation capacity of the network.

It should be apparent that the choice between an odd and even distribution is a binary one. As we are alternating nodes that perform Bell State Measurements with nodes that generate entanglement, the choice is in fact the operation that the first node will perform: a BSM or e-pair generation. Even though the difference between odd and even distribution is simple, they are not equivalent. As we can observe in Fig. 3b and d, in the cases where the number of intermediary nodes is even, there is indeed no difference in the number of BSM performed in total. However, for an odd number of intermediary nodes, it is preferable to perform an odd distribution, as it would imply one less BSM than the even distribution. This would increase the success rate of the distribution. In the following section, we will explore how these concepts can be applied for a crossbar quantum network, and how to find the optimal distribution scheme for any particular input–output configuration of the crossbar.

The principles of even and odd distributions can be extended and applied within the context of a crossbar architecture as well. For the sake of simplicity of operations, we will consider an $N \times N$-sized crossbar, without loss of generality. First, we need to define an operational cycle of the crossbar quantum network. Similar to a classical crossbar, a cycle is represented by the basic unit of time during which information transfer operations are executed. Within each cycle, we can define a tuple of input–output pairs, that describe the way quantum information flows through the crossbar during that cycle.

**Figure 4:**
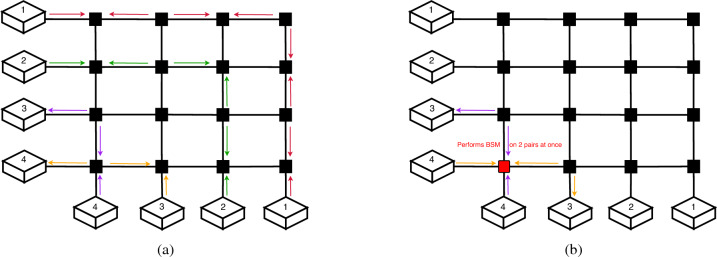
(**a**) is an example entanglement distribution on a $4 \times 4$ crossbar where we use the following boolean values to describe the distributions: [*false*, *false*, *true*, *true*]. In (**b**), there is an example of a distribution where the used distribution scheme does not work. Since the (3, 4) route uses an odd distribution, and the (4, 3) route uses an even distribution, they would end up using the same node to perform the Bell State Measurement. This would not lead to the desired entanglement distribution.

We will use the following numbering convention for input and output nodes of a crossbar. The input nodes (on the left side of the figures) are numbered starting from 1, top to bottom. The output nodes (at the bottom of the figures), are numbered starting from one, right to left. This convention is important as it will allow us to easily determine the routes which intersect based on the input and output indices. In order to better understand the tuple of input–output pairs, take for example the crossbar in Fig. 4a, for which we would have the following tuple that describes the flow: [(1, 1), (2, 2), (3, 4), (4, 3)]. We may take each input–output pair, and assign a boolean value to it - whether it performs an odd distribution (true), or an even distribution (false). It should be emphasized that the selected assignment convention was adopted arbitrarily and does not carry any inherent significance. An alternative convention could have been equally valid.

As we can see in Fig. 4b, the choice of whether or not to perform an odd distribution—which would be preferable due to the lower number of BSMs that it would imply, is not trivial. If we assign an odd distribution for every pair, we end up in a state where a single node would need to simultaneously perform a BSM for two separate incoming pairs of particles. This would not lead to the desired effect, as it would not swap the entanglement in the way that was intended. Therefore, we need to find a way to assign the routes an even or odd distribution, in a way such that a node only performs a BSM for one pair at a time.

### Optimal routing scheme for entanglement distribution in crossbar networks

As there are only two possibilities for each input–output pair, it might seem feasible to try out all the possible combinations for fairly small crossbar sizes. Such an approach would be faced a problem - how to determine if a combination is correct. In order to solve this, we need a way to determine whether or not two input–output pairs intersect, and, more importantly, what combinations does their intersection allow.

In order to determine whether or not two input–output pairs intersect, we need only to consider the tuple that describes the flow. For every input–output pair, we will consider all other pairs that have the input index larger than the current one. By looking only at the routes that have their input index higher, we are essentially looking at routes that start from a lower position in the crossbar. Two routes can intersect if the route that starts lower has its’ destination further right than the one that starts higher. Effectively, this means that if there are any pairs that have their input index higher than the one we are currently assessing, and have their destination index lower, these two routes intersect, per this numbering convention. In order to efficiently determine which routes intersect, we may sort the tuple by their input index. Afterwards, we start traversing the tuple, and for each pair, we will look at all the next pairs and perform this check. This intersection check would still have an algorithmic complexity of ${\mathcal {O}}(n^2)$, larger than the complexity for sorting the tuple. Note that we only performing the lookup for larger indices, as it is sufficient to detect the intersection once. By looking at all indices, we would detect intersections two times: once from the route that starts above, and once for the route that starts below.

**Figure 5:**
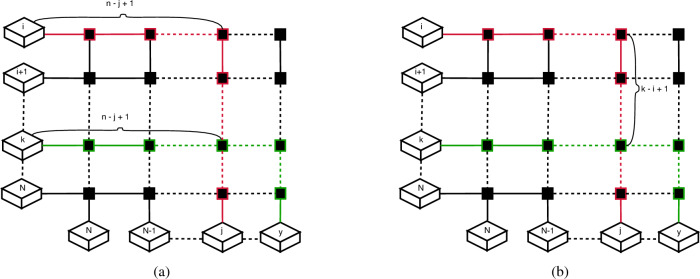
In (**a**) it can be observed that the number of nodes, along both routes, in a horizontal path, is equal to the total number of nodes, minus the destination of the uppermost route. We need to count one extra node, due to the fact that we consider the input node as part of the topology as well. In (**b**), along a vertical path, the uppermost route has to travel the difference between both input nodes. The node where the path switches should not be counted twice, that is why we subtract 1 from that part of the equation.

Determining whether or not two routes intersect is not enough however, to be able to check if a boolean combination would satisfy the conditions we imposed earlier. We also need to check what kind of route assignations are possible for the two intersecting routes. In order to determine this, we would need to determine for each route the respective parity of the intersection node. Let us consider two input–output pairs (*i*, *j*) and (*k*, *y*), with $i < k$ and $j > y$. Based on what we have established, these two routes intersect, and the first route has its input node above the other, as it can be observed in Fig. 5a. The intersection node parity for the (*i*, *j*) route can be calculated using the following formula: $(n-j+1+k-i)\ mod\ 2$. There are two parts of this formula. The $n-j+1$ is the number of nodes along the horizontal axis, followed by $k-i$, which is the number of nodes along the vertical axis. The parity for the (*k*, *y*) route has a simpler formula: $(n-j+1)\ mod\ 2$, which is just the number of nodes along the horizontal axis.

Based on the parities for the intersection node, we can impose some constraints upon the boolean values assigned for each route. In order to formally define these constraints, we will consider the following convention: the boolean variable *i* will be uniquely assigned to route (*i*, *j*), and will determine whether it will use an even or odd distribution, as previously stated. There are 4 possibilities regarding the parities for the intersection node. If for both routes the node is even, this means that they cannot both use an odd distribution at the same time. This constraint translates to $(i \vee k)$. If for route (*i*, *j*) the node is even, and for (*k*, *y*) odd, the first route cannot be odd while the second is even. This constraint can be written as $(\lnot i \vee k)$. The reverse is true, if for the first route the node is odd, and for the second even, the constraint is $(i \vee \lnot k)$. If for both routes the node is odd, both cannot use an even distribution, meaning that the constraint is $(\lnot i \vee \lnot k)$. These constraints need to be satisfied for all routes simultaneously - conjoined with an $\wedge$. Thus, we have a formula in the conjunctive normal form (CNF).

Having an array of booleans and a CNF formula means that this problem falls in the category of 2-satisfiability (2SAT) problems^56^. However, it is not enough to know if an input–output pair tuple is satisfiable, we also need to know the boolean configuration which satisfies the constraints we have imposed. Furthermore, we want optimality in terms of the lowest numbers of Bell State Measurements performed. Thus, we want as many odd distributions as possible. This means that we want a boolean configuration which satisfies the constraints, and has the largest amount of true values.

In canonical form, the problem which we want to solve can be expressed as follows

1 $$\begin{aligned} \begin{aligned} \text{Let } {\textbf{d}} = [d_1, d_2, \ldots , d_n] \text{ be a boolean vector}&\text{ where } d_i \in \{0, 1\} \text{ for all } i = 1, 2, \ldots , n&\\ \text{find } {\textbf{d}} \text{ that maximizes }&{\textbf{d}}^\top {\textbf{d}}&\\ \text{subject to }&(i \vee k) \text{ if intersection 1 is even for both or }&\\&(\lnot i \vee k) \text{ if intersection 1 is odd-even or }&\\&(i \vee \lnot k) \text{ if intersection 1 if even-odd or }&\\&(\lnot i \vee \lnot k) \text{ if the intersection 1 is odd for both}&\\&and&\\&(i \vee k) \text{ if the intersection 2 is even for both or }&\\&(\lnot i \vee k) \text{ if intersection 2 is odd-even or }&\\&(i \vee \lnot k) \text{ if the intersection 2 if even-odd or }&\\&(\lnot i \vee \lnot k) \text{ if intersection 2 is odd for both}&\\&and \ldots&\end{aligned} \end{aligned}$$

where all conditions are imposed by the way the routes intersect, as described before.

It is noteworthy that the trivial situation where we just want a configuration which satisfies the constraints is easily solvable in linear time by using a directed graph representation of the constraints, the situation where we want to maximize the number of true values is known in literature as a MAX-SAT problem, and its variants, like MAX-2SAT, are NP-hard. Algorithms based on branch-and-bound methods are known to solve MAX-2SAT problems, however, for large problem sizes, the solution may not be exact^57^.

## Simulation results and scalability analysis

In Algorithm 1 we present the constraint-building process for finding a boolean configuration which allows concurrency-free entanglement transmission in the crossbar quantum network, by solving the optimization problem described in Eq. 1. We consider a solver object which provides an API for adding constraints, and for solving the problem. The optimality of the solution, in terms of the number of true values, is depending on the solver used, and not on the constraints imposed. As such, the hereby presented algorithm is generally applicable.

**Algorithm 1:**
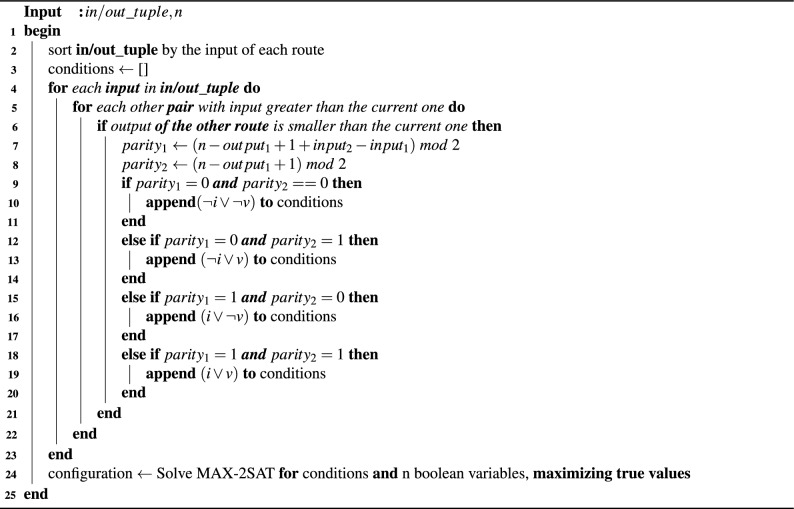
Optimal distribution configuration over the crossbar

**Figure 6:**
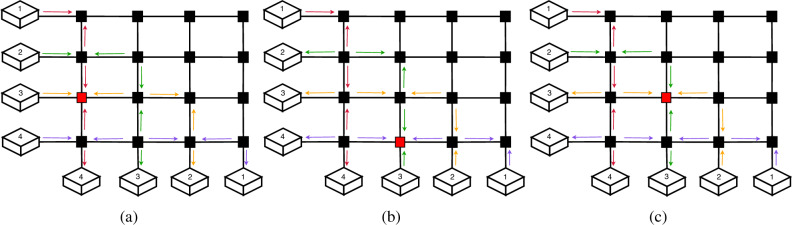
With an input–output configuration of [(1, 4), (2, 3), (3, 2), (4, 1)], it is impossible to find a configuration that wouldn’t result in a double measurement in at least one node. In (**a**), we can observe how, if routes 2 through 4 use an even distribution, the path for route 1 would have 3 consecutive nodes where BSMs are performed by the other routes. In (**b**), route 4 has 2 consecutive nodes which are used for BSM by routes 2 and 3. In (**c**), route 3 has two consecutive nodes used, by routes 1 and 2.

Using the previously presented algorithm, we have simulated all the possible input–output configurations for crossbars up to an $10 \times 10$ size (the simulations were performed on a machine equipped with an AMD Threadripper 5975WX, 32 cores and with 256GB RAM). Since the number of possible, distinct configurations is $N! - 1$, where N is the size of the crossbar, simulating crossbars of larger size would have been an increasingly difficult task.

In the simulation results, there are input–output configurations which are not satisfiable, such as the one in Fig. 6. This means that there is no way to distribute entanglement without having one or more nodes having to perform simultaneous BSMs on more than a pair. Such configurations all follow the same base pattern, which was first found for the $4 \times 4$ crossbar. We can observe in Fig. 6, how there is no configuration possible where we can avoid having to measure multiple pairs in the same node.

**Figure 7:**
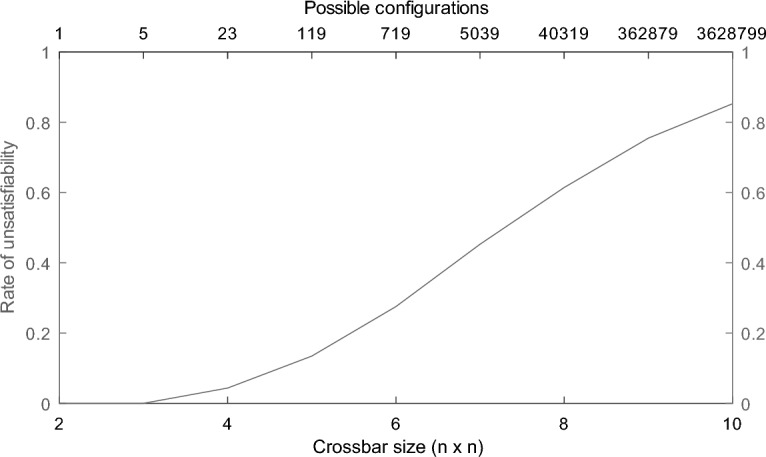
The rate of unsatisfiable configurations in relation to the size of the crossbar. For crossbars smaller than $4 \times 4$, all configurations are satisfiable. The $4 \times 4$ crossbar has a single unsatisfiable configuration out of the 23 total possible. The rate of unsatisfiability greatly increases afterwards, reaching almost 50% for a $7 \times 7$ crossbar.

Furthermore, it can be observed in Fig. 7 that the incidence of unsatisfiable configurations greatly increases. Even going from a crossbar of size $4 \times 4$ to $7 \times 7$, the rate of unsatisfiable configurations increases tenfold, going from approx. 0.043 to approx. 0.45. Thus, it can be safely concluded that the usability of non-blocking entanglement distribution algorithms that make use of alternating entanglement swapping patterns are not feasible for crossbars of larger size.

### Overcoming crossbar size limitations

The consequence of this finding is that, even though a crossbar quantum interconnection constructed in the manner described would indeed transfer entanglement within the minimal and deterministic latency, and offer non-blocking communication, it cannot fulfill the basic purpose of the classical crossbar interconnection, that is to offer a channel from every input to every output. If the aim is, however, to provide non-blocking communication with low, but not minimal latency, a multi-stage network, using crossbars up to size $3 \times 3$ would be feasible, as there are no configurations where those have concurrency over an intersection node.

**Figure 8:**
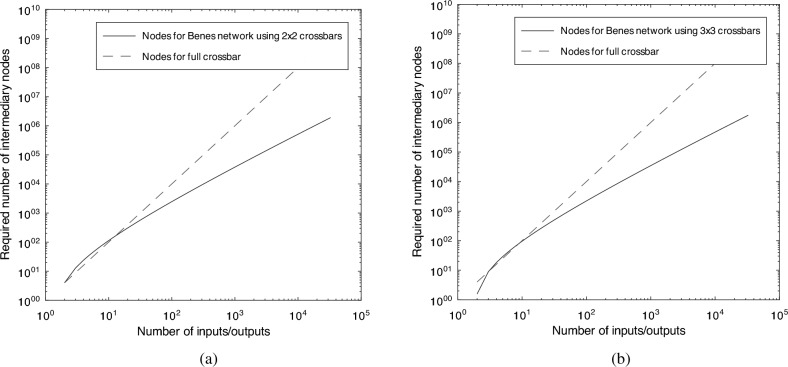
Logarithmic plot of the number of intermediary nodes placed within $2 \times 2$-sized crossbars within a Beneš network. It can be observed that for relatively low number of inputs/outputs (approx. 20), a full crossbar would have required less intermediary nodes. However, this would have come at the cost of the impossibility of performing efficient entanglement distribution for certain input–output configurations.

For example, we could distribute entanglement to any number of quantum nodes, in a non-blocking manner, by using crossbars quantum networks of size $2 \times 2$, through a Beneš network. Such a setup would require $N * \log _2{N} - N/2$ intermediary crossbars, totalling $4 N * \log _2{N} - 2 N$ intermediary nodes. Alternatively, if we were to use a similar scheme, but use crossbars of size $3 \times 3$, we would require $\frac{2}{3} * N \log _3{N} - \frac{N}{3}$ crossbars. Since every such crossbar would have 9 intermediary nodes, this leads to a total of $6 N * \log _3{N} - 3 N$. We can observe how each of these two solutions compares to a full crossbar between all $N \times N$ nodes in Fig. 8.

Alternatively, as the Beneš network is a special case of the Clos network, where the crossbars have a $2 \times 2$ size, we may use crossbars of size $2 \times 3$, $3 \times 2$, or $3 \times 3$, to construct a non-blocking multi-stage interconnection network, to distribute entanglement.

## Conclusions and future outlook

This paper introduces an approach that addresses a problem not been previously investigated, efficiently distributing entanglement through a crossbar quantum interconnection network. Efficient distribution of entangled particles plays a pivotal role in enhancing quantum communication, where entanglement is a building block for secure data transfer, either through QKD or teleportation. Moreover, in quantum computing, efficient distribution of entanglement is essential for scaling up systems and maintaining coherence across qubits, thereby improving computational capabilities.

We have proposed two different distribution strategies for any route within the crossbar: even distribution and odd distribution. These distribution strategies ensure minimal latency for the entanglement distribution operation, though the odd distribution has a greater success rate, due to one less Bell State Measurement being performed. Choosing the optimal configuration for an operation cycle of the crossbar, is therefore a problem of optimizing the number of odd distributions performed, while satisfying the constraints being placed between individual routes. Namely, intermediary nodes may perform only one BSM at a time. It should be noted that this way of distributing entanglement is not the only one possible, as we are imposing a harsh condition upon the qubits that we are transmitting - they do not remain coherent for more than a crossbar cycle. We are imposing this without losing generality, as operating with coherence times lower than this would be impossible, as they would not even survive the transmission within the links of the crossbar. Thus, protocols where the qubits can be stored within the nodes for multiple cycles can be an interesting research direction.

We have analyzed the capabilities of the distribution strategies over the crossbar quantum network, computing the optimal distribution strategies for all possible scenarios for crossbars up to size $10 \times 10$. We have found that there are input–output configurations where it is not possible to distribute entanglement in a non-blocking fashion using the proposed distribution strategies, if the crossbar has a size greater than $3 \times 3$. We have concluded that it is possible, albeit not with minimal latency, to construct a non-blocking, scalable, multistage network using crossbars of smaller sizes, which do not exhibit these problems.

As future work, we propose an analysis of optimality in terms of expected time of completion of the entanglement distribution process. Another proposed research direction is entanglement distribution in blocking interconnection networks. Another proposed analysis involves determining the best distribution strategy for qubits that can be stored at intermediary nodes for an unspecified duration in a quantum memory.

## Data Availability

The datasets generated during the current study and the complete mathematical calculus will be made available from the corresponding author on reasonable request.
